# Evaluating the costs of adverse drug events in hospitalized patients: a systematic review

**DOI:** 10.1186/s13561-024-00481-y

**Published:** 2024-02-08

**Authors:** Maxime Durand, Christel Castelli, Clarisse Roux-Marson, Jean-Marie Kinowski, Géraldine Leguelinel-Blache

**Affiliations:** 1 Department of Pharmacy, Nîmes University Hospital, Univ Montpellier, Nîmes, France; 2 Department of Law and Health Economics, Faculty of Pharmacy, Univ Montpellier, Montpellier, France; 3 Department of Innovation, Communication and Market, Univ Montpellier, Montpellier, France; 4 Department of Clinical Research, AESIO SANTE Méditerranée Beau Soleil Clinic, Montpellier, France; 5 Desbrest Institute of Epidemiology and Public Health, Univ Montpellier, INSERM, Montpellier, France

**Keywords:** Systematic review, Adverse drug events, Costs, Health economics, Pharmacovigilance

## Abstract

**Background:**

Adverse drug events (ADEs) are not only a safety and quality of care issue for patients, but also an economic issue with significant costs. Because they often occur during hospital stays, it is necessary to accurately quantify the costs of ADEs. This review aimed to investigate the methods to calculate these costs, and to characterize their nature.

**Methods:**

A systematic literature review was conducted to identify methods used to assess the cost of ADEs on Medline, Web of Science and Google Scholar. Original articles published from 2017 to 2022 in English and French were included. Economic evaluations were included if they concerned inpatients.

**Results:**

From 127 studies screened, 20 studies were analyzed. There was a high heterogeneity in nature of costs, methods used, values obtained, and time horizon chosen. A small number of studies considered non-medical (10%), indirect (20%) and opportunity costs (5%). Ten different methods for assessing the cost of ADEs have been reported and nine studies did not explain how they obtained their values.

**Conclusions:**

There is no consensus in the literature on how to assess the costs of ADEs, due to the heterogeneity of contexts and the choice of different economic perspectives. Our study adds a well-deserved overview of the existing literature that can be a solid lead for future studies and method implementation.

**Trial registration:**

PROSPERO registration CRD42023413071.

## Background

Drugs have become indispensable therapeutic tools and are a key factor in improving the quality of life and life expectancy of many people, but their use (i.e. prescribing, dispensing, preparation and administration) remains complex and a potential source of adverse events. As stated in another study, the life expectancy itself also has an important impact on a country’s economy [1]. The identification, characterization and understanding of these adverse events has been continuously improving since the late 90s [2]. The World Health Organization defines an Adverse Drug Event (ADE) as an unfavorable consequence involving a drug, whether preventable (e.g., the result of a Medication Error (ME)) or not (e.g., an Adverse Drug Reaction (ADR)) [3]. Unsafe medication practices and MEs are a leading cause of injury and avoidable harm in health care systems across the world. Most of ADEs do not result in significant harm for patients, but some drugs can lead to prolonged hospital stays, complications and disabling sequelae or death. All this inexorably leads to an increase in the cost of therapy, surplus that could be used to fund other health needs. Numerous national surveys around the world have described and quantified ADEs as a public health issue. However, only a few studies have focused on methods for evaluating the costs generated by ADEs, even though the WHO estimated in 2017 that the annual cost generated by MEs was 42 billion dollars worldwide. This represents nearly 1% of all health care expenditure worldwide [4]. ADEs represent an important item of expenditure for healthcare systems and their prevention could be associated with significant cost savings. There is no consensus on how to evaluate the costs of ADEs. In similar clinical settings, there is no evidence to suggest that one approach should be used over another, and this is where the difficulty lies in establishing a precise method for assessing the real cost of ADEs to compare different outcomes.

Our systematic review aims to assess the different methods used for evaluating the economic impact of adverse drug events in hospitalized patients, in order to implement a better management system for stakeholders.

## Methods

A systematic literature review was conducted to identify recent studies that assessed the cost of ADEs. The methodological data of this systematic review is in alignment with the Preferred Reporting Items for Systematic Reviews and Meta-Analyses (PRISMA) statement recommendations updated in 2020 [5]. The protocol of this systematic review is registered in Prospero under the ID: CRD42023413071.

### Literature search

A systematic search was conducted on three databases Medline (Pubmed), Web of Science and Google Scholar to identify studies presenting the nature, value and methods of assessing the costs of ADEs. The searches included studies published between 1st January 2017 and 1st June 2022. Search algorithms are as follows:

- Medline: (((pharmacoeconomic*[Title]) OR (economic*[Title]) OR (cost*[Title])) AND ((adverse drug event*[Title]) OR (adverse drug reaction*[Title]) OR (medication error*[Title])))
- Web of science: (((TI = ((pharmacoeconomic* OR economic* OR cost*) AND (adverse drug event* OR adverse drug reaction* OR medication error*)))))
- Google Scholar: allintitle: pharmacoeconomic OR economic OR costs AND “adverse drug events” OR “adverse drug reaction” OR “medication error”

The search associated cost-related keywords with the different types of ADEs.

### Study selection and exclusion criteria

Initially, two researchers (MD and GLB) screened by hand the title and abstracts. Systematic reviews and publications not presented as original articles (letters, congress abstracts, thesis) were excluded. Only literature published in English and French was included. Titles and abstracts were read to exclude off-topic publications that did not address costing and ADEs (in its broadest definition, including ADRs and MEs) and which did not include inpatients. The articles for which the full text was unavailable were also excluded. The publications were not selected according to the type of study, their geographical origin, or the socio-demographic or clinical characteristics of the patients included, given the limited number of articles published on this subject. Then, after sourcing the articles, the full texts were read and analyzed independently by two researchers (MD and GLB) to identify each methodology used to evaluate the costs associated with ADEs.

### Data extraction

The general data was extracted from the articles included in the review to characterize the studies. Firstly, the clinical characteristics were retrieved: general data (authors, publishing year), geographical data (country, clinical area), method and study design, time horizon, population settings, suspected drugs and type of ADEs. Then, the economic settings as the type of cost analysis, cost components assessed and results. Two authors (MD and GLB) have assembled a table to aggregate the extracted data. This dataset has been adapted throughout the work to clarify the information comprised in the publications. Table 1 presents the terminology used to define, classify and harmonize the different components of a cost (direct, indirect or opportunity) [6].

**Table 1:** Terminology used to describe inputs of calculations according to Patel et al. [6]

**Classification of costs included in calculations**	**Glossary of terminology**
**Pharmaceutical**	To include the terms below
**Medication costs**	Raw pharmaceutical cost of the drug involved
**Drug monitoring**	Any procedures/investigations required to maintain a drug within its therapeutic range
**Investigations**	Any tests undertaken with therapeutic intent
**Laboratory**	To include all pathology services
**Radiology**	To include all imaging modalities
**Procedures**	Any examination or intervention with a therapeutic intent
**Diagnostic**	Examination of interventions to determine a condition or disease
**Surgical**	Interventions that involve break of the skin with therapeutic intent
**Therapies**	Synonym of procedure
**Labour**	The sum of all wages paid to employees
**Pharmacist**	Wages paid to pharmacists
**Physician**	Wages paid to doctor/physician
**Services**	Cost of providing a particular facility for a patient’s care
**Outpatients care**	Cost of hospital-based clinics and associated investigations and treatments
**Home visits**	Cost of patients care in their own home
**Transport/travel**	Cost of patient transport
**Ambulatory care**	Cost of hospital visit short of an admission
**Nursing facility stay**	Cost of a stay at a nursing facility
**Emergency department visit**	Cost of a visit to the emergency department
**Intensive care stay**	Cost of admission to an intensive care unit
**Infrastructure**	Cost of physical facilities for delivering healthcare
**Bed**	Cost of providing physical facilities and care surrounding an individual patient’s treatment
**Overheads**	Operating expenses not directly related to a single patient admission (e.g., a fixed cost)
**Equipment**	Cost of consumables used to deliver patient care
**Medical supplies**	Cost of consumables used to deliver patient care
**Hospital admission**	Sum of all variable costs that can be attributed to a single patient
**Patient**	Any cost borne by the consumer of the service
**Cost of permanent harm**	Estimation of the cost of living with any lasting consequence of a medication error
**Income**	Loss of patient income due to increased time spent in hospital as a result of medication error
**Economic**	Accounting cost plus opportunity cost
**Opportunity costs**	The cost of an alternative that must be forgone in order to pursue a certain action

### Qualitative appraisal

Authors used the Consolidated Health Economic Evaluation Reporting Standards (CHEERS) checklist, version 2022 [7]. Quality was independently assessed by two authors (MD and GLB). A score was attributed to each article. When the information was available and well reported, a score of 1 point was assigned. If the information was incomplete, 0.5 point was assigned. Finally, 0 point was assigned when the information was not present. The final scores were converted into a mark ranging from 0 to 1. A high mark corresponded to a higher reporting quality. All discrepancies in the assessment were resolved by consensus between two authors (MD and GLB). The discount rate was considered relevant only for time horizons above 1 year.

Data analyses were performed using Microsoft Excel 365 (Microsoft Corporation, Redmond, WA, USA). All costs were converted into Euro (€) according to the closing exchange rate from 07/09/2022 and were not adjusted for inflation or discounted.

### Ethical aspects

This is a secondary literature review; no ethics committee approval was required.

## Results

### Search results

**Fig. 1:**
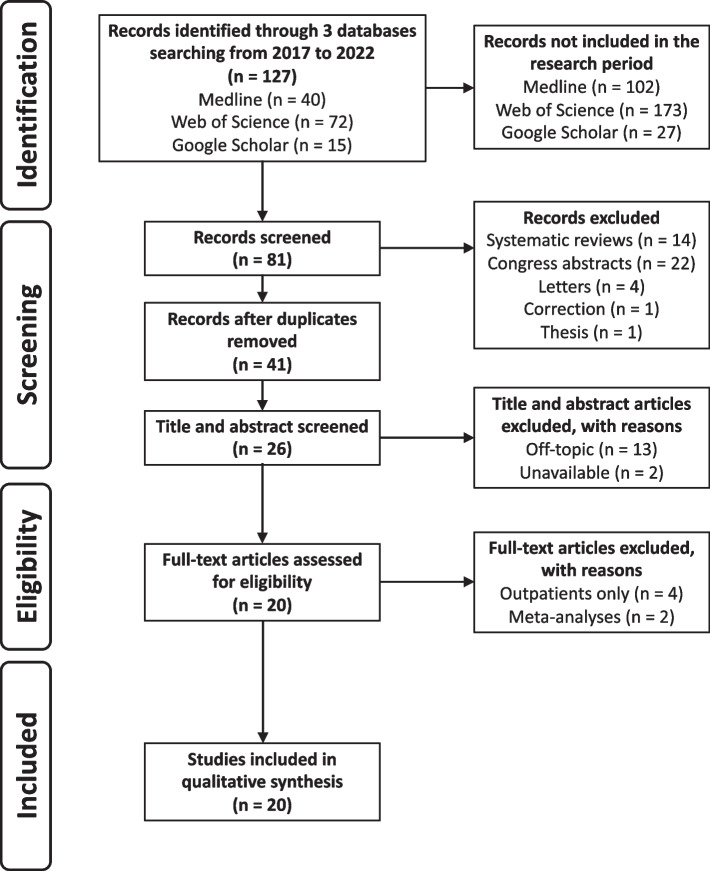
Flowchart of study selection

The initial search identified a total of 127 potentially relevant publications. After duplicates removal and after applying the exclusion criteria to the titles and abstracts, only 26 publications remained. The full text reading resulted in a final selection of 20 articles used for the review. The selection is shown in Fig. 1.

### Characteristics of the studies

A methodology summary of the included studies is presented in Table 2. Seven publications were from USA, two from Japan, South Africa, and Sweden, respectively and one from Canada, France, India, Iran, Korea, Malaysia, Taiwan, respectively. None of the studies were published in 2020. Fifteen studies focused only on secondary and tertiary care (inpatients), while five focused on primary, secondary, and tertiary care (both inpatients and outpatients). All the publications included were observational studies, 90% were retrospective and 60% were multi-centric. Three studies were based on pharmacovigilance databases (15%) [8–10]. More than half of the studies included all ADEs (*n* = 11, 55%), while eight focused on ADRs and only one on MEs. Twelve studies (60%) were not focused on a specific therapeutic group. Among the studies which analyzed a specific therapeutic group, the cost of ADEs caused by anti-infective drugs (*n* = 3, 15%) [11–13] or painkiller drugs (*n* = 2, 10%) [14, 15] were the most evaluated. Most of the studies did not assess a specific ADE (*n* = 18, 90%). Only one focused on cutaneous ADEs [11] and another on gastrointestinal ADEs [14]. Two studies (10%) evaluated the costs of ADEs in geriatric population [16, 17] and two (10%) in pediatric population [12, 18]. Eight (40%) studies included ≤ 1000 patients and 11 (55%) included > 1000 patients. One study did not specify the number of patients included and expressed its results in number of events [8].

**Table 2:** Studies calculating the cost of adverse drug events, clinical data

**N°**	**First author (year) (ref)**	**Country**	**Clinical area**	**Study design**	**Number of patients**	**Type of ADE**	**Population Settings**	**Suspected drug(s)**
1	Katsuno et al. (2021) [19]	Japan	Inpatients and outpatients	- observational - monocentric - retrospective	359	ADE	Patient aged > 1 year	All (except experimental drugs)
2	Trumbo et al. (2021) [8]	USA	Inpatients and outpatients	Pharmacovigilance database - observational - multicentric - retrospective	NK	ADE	All patients	Intravenous iron
3	Iwasaki et al. (2021) [18]	Japan	Inpatients (tertiary care hospital)	- observational - multicentric - retrospective	907	Preventable ADE	Patients < 15 years and hospitalised in any unit and patients over 15 years old hospitalised in paediatric units	All prescribed medication
4	Tissot et al. (2021) [9]	France	Inpatients and outpatients	Pharmacovigilance database - observational - multicentric - retrospective	1185	Serious ADR	All patients except patients with serious congenital anomalies related	All
5	Maity et al. (2021) [10]	Canada	Inpatients	Pharmacovigilance database - observational - multicentric - retrospective	63 069	ADR	All patients	Remicad® and Humira® (biosimilars were not included)
6	Knight et al. (2019) [11]	South Africa	Inpatients (tertiary care hospital)	- observational - monocentric - retrospective	89	ADR	All patients with drug sensitive tuberculosis with a suspected cutaneous adverse drug reaction to first line anti-tuberculosis drugs	First line anti-tuberculosis drugs
7	Boostani et al. (2019) [20]	Iran	Inpatients (tertiary care hospital / internal medicine unit)	- observational - monocentric - prospective	100	ME	Patients hospitalised in internal medicine ward	All
8	Riaz et al. (2019) [16]	USA	Inpatients (tertiary care hospital)	- observational - multicentric - retrospective	3 832 322	ADE	Age 65 and above	All
9	Beck et al. (2019) [12]	USA	Inpatients (emergency room and tertiary care)	- observational - monocentric - retrospective	430	ADR	Patients under 21	Antibiotics
10	Lee et al. (2019) [21]	Korea	Inpatients (emergency room and tertiary care)	- observational - multicentric - retrospective	903	ADR	All patients	All
11	Liao et al. (2019) [17]	Taiwan	Inpatients (tertiary care hospital)	- observational - monocentric - retrospective	2 393	ADR	Age 65 and above	All
12	Pok et al. (2018) [14]	Malaysia	Inpatients (tertiary care hospital/ rheumatology unit)	- observational - multicentric - retrospective	634	ADE	Patient hospitalised in the rheumatology department with osteoarthritis or rheumatoid arthritis	NSAID
13	Schnippel et al. (2018) [13]	South Africa	Inpatients	- observational - multicentric - retrospective	12 527	ADR	Multi drugs resistant (+ rifampicin) tuberculosis patients	Drugs used in rifampicin-resistant tuberculosis
14	Shafi et al. (2018) [15]	USA	Inpatients (tertiary care hospital/ Surgery and endoscopy unit)	- observational - multicentric - retrospective	135 379	ADE	Age 18 and above (patients having surgery or endoscopy)	Opioids
15	Kurle et al. (2018) [22]	India	Inpatients (tertiary care hospital / dermatology unit)	- observational - monocentric - prospective	55	ADR	All patients except patients using alternative therapies	All
16	Slight et al. (2018) [23]	USA	Inpatients (tertiary care hospital)	- observational - monocentric - retrospective	40 990	Preventable ADE	All patients	All
17	Gyllensten et al. (2017) [24]	Sweden	Inpatients and outpatients	- observational - multicentric - retrospective	4 970	ADE	Age 18 and above and living in the Östergötland county, Sweden	All
18	Natanaelsson et al. (2017) [25]	Sweden	Inpatients and outpatients	- observational - multicentric - retrospective	5 025	ADE	Age 18 and above and living in the Östergötland county, Sweden	All
19	McCarthy Jr. et al. (2017) [26]	USA	Inpatients (tertiary care hospital)	- observational - monocentric - retrospective	3 521	ADE	All patients	All
20	Spector et al. (2017) [27]	USA	Inpatients (tertiary care hospital)	- observational - multicentric - retrospective	85 338	ADE	Age 18 and above	Anticoagulants and Hypoglycaemic agents

### Cost analysis

As illustrated in Table 3, all of the 20 selected studies conducted cost analyses. Half (50%) of them had a time horizon ≤ 1 year, with an average of 30 months and a median of 12.5 [7.5–48.0] months. The inputs and methods used to estimate or calculate costs generated by ADEs had a high degree of heterogeneity. Synthesized in Table 4, 15 studies directly calculated the cost of ADEs while five studies estimated this cost based on external data or previous studies.

**Table 3:** Studies calculating the cost of adverse drug events, economic data

**N°**	**First author (year) (ref)**	**Costs Settings**	**Perspective**	**Time horizon**	**Quality score**	**Individual cost (per patient)**
1	Katsuno et al. (2021) [19]	**Direct costs (micro-costing):** Each clinic/technical fee including medical supplies and drugs	Public health insurance	6 months	0.74	€ 5 937
2	Trumbo et al. (2021) [8]	**Estimated direct costs:** Hospital admission costs	Hospital	6 years	0.69	NA
3	Iwasaki et al. (2021) [18]	**Estimated direct costs:** Daily hospital medical expenditure	Hospital	3 months	0.87	€ 6 034
4	Tissot et al. (2021) [9]	**Direct medical costs:** Hospital admission costs and additional items (daily supplements for extreme lengths of hospital stays, daily supplements for intensive care stay, expensive drugs or medical devices)	Public health insurance	4 years	0.67	€5 559 ± 8 448 median: €3 725 IQR: [401;114 576]
5	Maity et al. (2021) [10]	**Direct costs:** Direct healthcare costs (national average cost of hospitalisation for poisoning/toxic effect of drug category for different age groups) **Indirect/Opportunity costs:** Potential loss of income over a lifetime with human capital approach (calculated for patient aged between 19–64; ages below 19, only future forgone incomes were included) Lost productivity with friction cost approach	3 = Patient + Health system + Societal	4 years	0.92	Patient perspective: €451 (infliximab) €359 (adalimumab) Health system perspective: €230 (infliximab) €100 (adalimumab) Society perspective: €240 (infliximab) €110 (adalimumab)
6	Knight et al. (2019) [11]	**Direct costs:** Hospital costs (bed, food, basic nursing, infrastructure), average (mid-range) salary in each personnel category was applied accounting for the varying levels of attending staff, laboratory and radiology investigations, medications costs	Hospital	5 years	0.63	€5 831 CI_95%_: 5134–6527
7	Boostani et al. (2019) [20]	**Estimated direct costs:** Medication cost	2 = Patient, Health insurance	8 months	0.70	€7.46
8	Riaz et al. (2019) [16]	**Direct costs:** Hospital admission costs	Public health insurance (MEDICARE)	11 months	0.87	NK
9	Beck et al. (2019) [12]	**Estimated direct costs:** Emergency department visit + intensive care stay + hospital admission costs	Public health insurance (MEDICAID)	1 year	0.48	€456
10	Lee et al. (2019) [21]	**Direct medical costs (micro-costing):** Costs related to physician + medication cost + laboratory + radiology + hospital admission + surgical + medical supplies	Hospital	6 months	0.67	€1 881 incremental costs compare to before ADR
11	Liao et al. (2019) [17]	**Direct medical costs:** Hospital admission and medication costs	Hospital	6 years	0.70	€5 422.40 medical expenses incremental costs compare to non-ADR group control €1459.40 medication incremental costs compare to non-ADR group control
12	Pok et al. (2018) [14]	**Direct medical costs:** Hospital admission + outpatient care + surgical procedures + diagnostic investigations + treatment costs for managing upper GI adverse events	Hospital	2 years	0.67	€136.60
13	Schnippel et al. (2018) [13]	**Direct medical costs:** Medication cost + laboratory/investigation + outpatient care + hospital admission + medical supplies	Hospital	10 years	0.70	€380.17 incremental costs compared to no ADR
14	Shafi et al. (2018) [15]	**Direct medical costs:** Hospital admission costs	Health system	33 months	0.71	€8 225 incremental cost compared to no ADR
15	Kurle et al. (2018) [22]	**Direct costs:** Investigation + drugs + transport costs **Indirect costs:** Loss of income (patient + relatives)	Patient	1 year	0.70	€83.97
16	Slight et al. (2018) [23]	**Estimated cost of a preventable ADE in inpatients:** Bates 1997 [28]: Hospital admission costs Hug 2012 [29]: Hospital admission + overheads + capital costs **Opportunity costs:** Estimating the time and cost of responding to Clinical Decision Support alerts	Hospital	1 year	0.76	NK
17	Gyllensten et al. (2017) [24]	**Direct costs:** Outpatient care + home visits (nurse or physician) + hospital admission costs	Unspecified	3 months	0.76	€1 500 (diagnostic code method) €414 000 (unit cost method)
18	Natanaelsson et al. (2017) [25]	**Direct costs:** Outpatient care + ambulatory care + hospital admission + medication costs + Labour **Indirect costs:** Lost productivity/income	4 = Hospital, patient, public health insurance, employer	3 months	0.83	€505 (with €192 of indirect costs) (real costs) €1 631 (€410 of indirect costs) (propension scoring)
19	McCarthy Jr. et al. (2017) [26]	**Direct medical costs:** Hospital admission costs	Hospital	13 months	0.65	€2 271€ IQR: 981–13 455 (incremental costs)
20	Spector et al. (2017) [27]	**Direct costs:** Hospital admission/annual hospital costs (“production of hospital services) excluding physician wages	Hospital	3 years	0.67	Anticoagulant: €7 644 (acute myocardial infarction) to €12 849€ (pneumonia) Hypoglycaemic agent: €2 747 (heart failure) to €9 054 (major surgery)

*NK* Not known, *NA* Not applicable

**Table 4:** Classification and types of calculation

**Method of calculation**	**Nb of studies (%)**	**N° (**Table 3**)**
Studies directly calculating the cost of ADE	15 (75)	1, 4, 5, 6, 8, 10, 11, 12, 13, 14, 15, 17, 18, 19, 20
Studies estimating the cost of ADE through external data or studies	5 (25)	2, 3, 7, 9, 16
Number of direct costs considered in the calculation:		
1	10 (50)	2, 3, 5, 7, 8, 9, 14, 19, 20
2	1 (5)	11
≥ 3	9 (45)	1, 4, 6, 10, 12, 13, 15, 16, 17, 18
Only direct costs	16 (80)	1, 2, 3, 4, 6, 7, 8, 9, 10, 11, 12, 13, 14, 17, 19, 20
+ indirect costs	3 (15)	5, 15, 18
+ opportunity costs	1 (5)	16
**Micro-costing method** Real costs of exact resources consumed in the care of each patient	4 (20)	1, 6, 12, 18
**ADR vs. non-ADR method (propensity scores matching)** Costs difference between ADR patients and matched control (non-ADR patient)	3 (15)	11, 18, 19
**Gross-costing method** Assign average values from national administrative databases	2 (10)	5, 13
**Extended LOS attributable to preventable ADE method** Extra medical costs = Hospital admission x number of patients with preventable ADEs × estimated extended LOS	1 (5)	3
**Before vs. after ADR method** Difference between the total medical care cost of a patient 6 months before the ER visit for an ADR and 6 months after Expenses for a new diagnosis of diseases (other than those diagnosed during the control period) were not included	1 (5)	10
**Resource use method** 1. ADE identification: manual from medical records, causality assessments between potential ADEs and drug therapies with causality levels (definite/likely/possible) 2. Resource use identification: contribution to healthcare use: assessment of each ADEs contribution to resource use, with contribution levels (dominant/partly/less) 3. Proportion of costs from regional cost per patient register: ADE contribution dominant = full costs ADE contributed partly or less = cost for specific resources used for ADEs	1 (5)	17
**Proportion of registered costs method** 1. ADE identification: manual from medical records, causality assessments between potential ADEs and drug therapies with causality levels (definite/likely/possible) 2. Resource use identification: contribution to healthcare use: assessment of each ADEs contribution to resource use, with contribution levels (dominant/partly/less) 3. Proportion of costs from regional cost per patient register: ADE contribution dominant = full costs ADE contributed partly = $1 \!\left/ \! 2 \right.$ of costs ADE contributed less = $1 \left/ 3 \right.$ of costs	1 (5)	17
**Unit cost method** 1. ADE identification: manual from medical records, causality assessments between potential ADEs and drug therapies with causality levels (definite/likely/possible) 2. Resource use identification: contribution to healthcare use: assessment of each ADEs contribution to resource use, with contribution levels (dominant/partly/less) 3. Proportion of costs from national statistics: ADE contribution dominant = full costs ADE contributed partly = $1 \left/ 2 \right.$ of costs ADE contributed less = $1 \left/ 3 \right.$ of costs	1 (5)	17
**Diagnostic code method** 1. ADE identification with ICD codes indicating ADEs 2. Resource use identification: all resource use during the healthcare encounter assigned to the ADE 3. Estimating cost with regional or national registers: full costs, $1 \left/ 2 \right.$ costs, $1 \left/ 3\right.$ costs	1 (5)	17
**Main diagnosis method** 1. ADE identification: manual matching with main diagnosis and ICD codes 2. Resource use identification: all resource use during the healthcare encounter assigned to the ADE 3. Estimating cost with regional or national registers: full costs, $1 \left/ 2 \right.$ costs, $1 \left/ 3\right.$ costs	1 (5)	17

*LOS* Long of stay, *ICD* International code of disease

#### Direct costs

Among the studies included, 80% of cost analyses were based only on direct costs and did not assess indirect or opportunity costs. When direct costs were calculated, most studies (*n* = 10) considered only one direct cost, while one study reported two types of direct costs and nine studies included 3 or more direct costs (Table 3).

Only 2 studies considered non-medical costs. Knight et al. [11] included the cost of food served to the patients in their calculation and Kurle et al. [22] included the cost of patients’ transport to hospital.

#### Indirect / opportunity costs

Of the 20 selected studies, 4 included indirect costs in their calculations and opportunity costs. Indirect costs were mentioned by Maity et al. [10] including potential lost wages over a lifetime using the human capital approach through the patient perspective and loss of productivity using the friction cost approach through the societal perspective. Kurle et al. [22] only considered the loss of patient’s wages and his/her relatives’ while Natanaelsson et al. [25] consider the loss of productivity and income through patient’s and employer’s perspective. Slight et al. [23] was the only study to consider ADEs generated opportunity costs by estimating the time and cost of responding to clinical decision support alerts (time not used for another activities). No study investigated only indirect costs.

#### Economic perspectives

Five different perspectives were selected from the 20 publications (Table 3). Eleven studies (55%) were conducted from a hospital perspective, 8 (40%) from a health system and/or public health insurance perspective, 4 (20%) from a patient perspective, 1 (5%) from an employer perspective and 1 (5%) from a societal perspective. Boostani et al. [20] included both a patient and health insurance perspective, Maity et al. [10] included a patient, health system and societal perspective and Natanaelsson et al. [25] a hospital, patient, public health insurance and employer perspective.

#### Costs results

The main finding regarding the cost of ADEs is the significant heterogeneity of the measures used to report costs and the values obtained. Costs due to ADEs (per hospitalization) ranged from around €6 000 to €10 000 from a hospital, health insurance or health system perspective. Five studies reported incremental costs.

#### Economic methods

As illustrated in Table 4, 10 different methods for assessing cost of ADEs have been reported. Eleven studies detailed which method they used to calculate their costs. Natanaelsson et al. [25] described two methods, one that underestimated costs and one that overestimated them. Gyllensten et al. [24] described five methods, using different combinations of three calculation steps. Nine studies did not detail how they obtained their values.

### Quality appraisal of the included studies

The CHEERS V2022 checklist include 28 points, some were not applicable to all or part of the studies. This quality assessment resulted in a mean score of 0.72, with a median of 0.70 IQR [0.67–0.76] and a minimum score of 0.48 and a maximum of 0.92 (Table 3).

## Discussion

Adverse drug events (ADEs) are a significant concern within the healthcare system, rising many challenges to patient safety and healthcare costs.

Through the available information, this is the first study focused on identifying designs, omissions, and potential strategies for assessing the economic consequences associated with ADEs.

The costs of ADEs are of interest to political and social decision-makers as well as to the hospitals themselves.

One of the main findings of our systematic review is the heterogeneity among the methods of reporting costs across different studies and healthcare systems. This can be attributed to differences in healthcare infrastructures, patient populations, and the nature of ADEs.

Most of the studies evaluated direct medical costs (e.g., hospital admission costs) through a hospital perspective. Few studies had a wider perspective (e.g., societal or health system) and explored indirect / opportunity costs. However, in chronic or disabling diseases caused by ADEs, indirect costs may represent the largest share of the cost [30–32]. The lack of data needed to value indirect costs and the choice of economic perspective could be reasons for not valuing indirect costs [28]. The patient perspective and non-medical costs were not considered in most of studies whereas the out-of-pocket expenses for the patient or his/her relatives could be high in countries with little public health insurance. So, the health facilities’ or authorities’ perspectives were predominant because studies were conducted to help local or national health decision makers. In their review, Batel Marques et al. [28] noticed that the hospital perspective appears to be the privileged perspective for the identification of ADEs and their costs because of the easy access to a complete description of each case through administrative databases.

The high variation of the costs can be explained by the considerable methodological heterogeneity in the calculation of ADE costs between studies. On the one hand, micro-costing [11, 14, 19, 25] is a cost estimation method that provides accurate values, considering each input consumed, though collecting such detailed usage and valuation data requires significant time and human resources [33]. On the other hand, the ADR vs. non-ADR method [17, 25, 26] is better suited to very large cohorts and allows a more global approach. The matching of patients using propensity scores makes the method very robust for the calculation of incremental costs [34]. Micro-costing is known to underestimate costs due to lack of detail in the registers containing healthcare cost and in the information on lost productivity resulting from ADEs (e.g., sick leave). On the other hand, the ADR vs. non-ADR method may overestimate the costs if there are unmeasured confounding factors. Accordingly, these two methods are complementary.

Therefore, gross costing [10, 13] has several advantages which partly counterbalance the drawbacks of micro-costing. In terms of feasibility, because hospital cost data consists of aggregate data, its estimation can be done quickly. In terms of cost, this method is inexpensive as it largely relies on administrative databases. In this case, the results of the study are easier to generalize. There are several drawbacks as well, especially the lack of precision since a cost cannot be associated with a specific component of the hospital stay because of the aggregate data, which is examined at an overall level. With this method, differences in terms of consumption of resources (e.g. different inpatient profiles) are thus unknown and individual variations are not considered [35].

To investigate indirect costs, the human capital approach uses the amount of gross national product (GNP) per capita. It is straightforward to deduce the average amount of output per individual over a given time. This is a simple method of comparing similar events with each other but does not yield true indirect costs because the method does not consider the exact activity of the patient. Another method is to list as many additional costs associated with the disease as possible, based on the average salary for the patient's socio-professional category or the exact salary if available. This may also allow an assessment of the value of the time lost by the patient’s relatives, for example, in maintaining the patient’s home during his or her absence, for childcare or for travel. The evaluation of indirect costs is interesting because it considers the individual’s social role. Also, Patel et al. [6] similarly concluded for the costs of MEs that there was a general inconsistency of the method used by the researchers. The way similar calculations were performed varied considerably across studies as the nature, number of inputs and stages within a given calculation were not uniform. However, they did not focus on the different costing methodologies, but more on the nature of the costs included. Micro-costing was only specified where it was used.

Because the estimated costs for ADEs are highly affected by the choice of the costing methods, unclear results were obtained when computing the average value of an ADE cost. Hazardous extrapolations of ADE costs are still made using data prior to 2000 [23] and/or very short time horizons [18, 24, 25] and/or a single center or unit of care to make national projections [18], and/or in countries with very heterogeneous GNP and health systems. Assessing the cost of ADEs is therefore a very complex issue and this systematic review confirms that ADEs have a high economic impact. The diversity of drugs, population and methodologies analyzed have been reported too by Batel Marques et al. [28] as a source of high costs variations.

This review confirmed that the methods used for assessing costs were mainly based on the subjective judgement of the researchers. There is a need for a consensual method of calculating costs per perspective to monitor their evolution within a hospital, and also among hospitals or among countries. A standardized method should be established and followed for conducting and reporting cost analyses in order to improve the quality and comparability across studies. One of the proposed solutions is the development and implementation of a decision algorithm that considers the number of patients included, the data available to estimate costs, the time horizon and chosen perspective. A medico-administrative team composed of computer scientists and biostatisticians would allow the processing of data from very large cohorts.

### Limitations

Despite the strong insights retrieved from our systematic review, certain limitations are acknowledgeable. Heterogeneity among the included studies may have influenced the results. Moreover, the dynamic nature of healthcare systems and constantly evolving pharmacological environment ask for updates and reevaluations of the economic impact of ADEs. We did not only select original articles with a high quality according to the CHEERS V2022 checklist to assess the global quality of articles on this topic. Only studies published in English and French were included, which excluded several studies published in other languages that focused on local or national issues.

## Conclusions and perspectives

Assessing the cost of ADEs is therefore a very complex issue. Standardizing cost assessment methodologies and reporting practices should be a priority in order to optimize the homogeneity of findings and provide more accurate evaluations of the economic burden. Approaching the entanglements of ADE-related costs requires joint efforts from healthcare professionals, policymakers, researchers and even patients.

A universal method for assessing the ADE generated costs could be comprised of both direct and indirect costs evaluation, in order to have an overview on the situation in hand. Although resources consuming, the micro-costing method has the potential of exposing relevant details, even though an association with the ADR vs non-ADR method would be necessary to accurately characterize the cost generating factors.

Additionally, the introduction of modern technologies, such as data analytics and machine learning, highlights the potential for innovation in evaluating ADE generated costs.

Lastly, future studies should consider broader time horizons to efficiently assess the long-term impact of ADEs and, if possible, integrate the socio-economic context and the healthcare system’s specific layout.

## Data Availability

Data included in this paper will be made available on reasonable request from the corresponding author.

## Abbreviations

ADE: Adverse drug events
ME: Medication Error
ADR: Adverse Drug Reaction
PRISMA: Preferred Reporting Items for Systematic Reviews and Meta-Analyses
CHEERS: Consolidated Health Economic Evaluation Reporting Standards
GNP: Gross National Product
